# Editorial Department

**Published:** 1854-03

**Authors:** 


Dr. Daniel Brainard, of Chicago, is now in Paris. He is urging upon the French Academy the use of the lactate of iron as an injection in aneurismal tumors. At the same time French surgeons are discussing the use of the Proto-chloride in the same lesion. Probably there is not much preference between them, but it is also probable that this use of styptic injections into the pulsating or aneurismal tumors is a valuable addition to surgical treatment, and may, under favorable circumstances, do away with the necessity of tying large arteries.

Dr. Brainard reports a case in the Gazette des Hopitaux, of an aneurismal tumor of the orbit, treated by this method very successfully. Its rationale is simple, being merely the coagnlation of the blood in the tumor by the action of the iron, the consequent obliteration of the vascular net-work, and the final removal of the clot and tumor together by absorption. H.

